# *HTR1A, HTR1B, HTR2A, HTR2C* and *HTR6* Gene Polymorphisms and Extrapyramidal Side Effects in Haloperidol-Treated Patients with Schizophrenia

**DOI:** 10.3390/ijms21072345

**Published:** 2020-03-28

**Authors:** Mirko Grubor, Maja Zivkovic, Marina Sagud, Matea Nikolac Perkovic, Alma Mihaljevic-Peles, Nela Pivac, Dorotea Muck-Seler, Dubravka Svob Strac

**Affiliations:** 1 Faculty of Pharmacy and Biochemistry, University of Zagreb, 10 000 Zagreb, Croatia; mirko.grubor@gmail.com; 2 Department of Psychiatry, University Hospital Centre Zagreb, 10 000 Zagreb, Croatia; maja.zivkovic20@gmail.com (M.Z.); MarinaSagud@mail.com (M.S.); alma.mihaljevic.peles@mef.hr (A.M.-P.); 3 School of Medicine, University of Zagreb, 10 000 Zagreb, Croatia; 4 Laboratory for Molecular Neuropsychiatry, Division of Molecular Medicine, Rudjer Boskovic Institute, 10 000 Zagreb, Croatia; Matea.Nikolac.Perkovic@irb.hr (M.N.P.); npivac@irb.hr (N.P.); Dorotea.Mueck.Seler@irb.hr (D.M.-S.)

**Keywords:** schizophrenia, haloperidol, acute extrapyramidal side effects, serotonin receptors, gene polymorphisms, *HTR1B* gene polymorphism, akathisia

## Abstract

Schizophrenia is a serious, chronic psychiatric disorder requiring lifelong treatment. Extrapyramidal side effects (EPS) are common adverse reactions to antipsychotic medications. In addition to the dopaminergic system, serotonergic mechanisms, including serotonin (5-HT) receptors, might be involved in EPS development. This study aimed to examine molecular associations of *HTR1A, HTR1B, HTR2A, HTR2C* and *HTR6* gene polymorphisms with acute EPS in 229 male schizophrenia patients, following two weeks of haloperidol monotherapy. The Simpson–Angus Rating Scale for Extrapyramidal Side Effects (SAS), Barnes Akathisia Rating Scale (BARS) and Extrapyramidal Symptom Rating Scale (ESRS) were used to evaluate EPS severity. Genotyping was performed using real-time PCR, following extraction of blood DNA. Significant acute EPS appeared in 48.03% of schizophrenia patients. For the rs13212041 *HTR1B* gene polymorphism, affecting microRNA regulation of *HTR1B* gene expression, a higher frequency of TT carriers was found among haloperidol-treated patients with akathisia when compared to the group without akathisia symptoms. In comparison to C-allele carriers, patients carrying the TT genotype had higher akathisia severity, as determined by the SAS, BARS and ESRS scales. These molecular findings suggest potential involvement of 5-HT_1B_ receptors in akathisia development following haloperidol treatment, as well as possible epigenetic mechanisms of serotonergic modulation associated with antipsychotic-induced EPS.

## 1. Introduction

Schizophrenia is a serious, chronic psychiatric disorder, requiring lifelong treatment [1]. Haloperidol, a highly effective first-generation antipsychotic (FGA), is one of the most prescribed antipsychotics in Europe and the US, and it is often used in clinical trials as a comparator drug [2]. Due to its very strong antagonistic activity on dopamine D_2_ receptors of the mesolimbic dopamine pathway [3], haloperidol acts as a very potent antipsychotic agent, and it is included on the World Health Organization’s list of essential drugs [4]. However, like other FGAs, it is also associated with the development of both acute and long-term extrapyramidal side effects (EPS) [5], possibly due to blockade of the D_2_ receptor in the nigrostriatal pathway [6]. Specifically, it has been demonstrated that FGAs bind “tightly” to D_2_ receptors and dissociate slowly [7], with a D_2_ receptor occupancy of greater than 80% significantly contributing to the risk of EPS [8]. Other predictors of EPS include younger age, male gender, longer treatment durations, higher dosage, psychiatric diagnoses, such as mood disorder, and previous EPS history [8,9,10,11]. More recently, genetic factors have also been considered, including those related to the metabolism of antipsychotic drugs and free radical scavenging [12,13,14], as well as variants in genes coding for various components of the dopaminergic system [15,16,17].

EPS are well-known and common antipsychotic-induced movement disorders [18]. They include acute EPS, such as akathisia, acute dystonia and parkinsonism, which may occur within days or weeks of initiating treatment, as well as late-onset EPS, such as tardive dyskinesia that develop months or years after the antipsychotic therapy [19]. These serious and debilitating side effects often lead to reduction of patient compliance or even discontinuation of therapy and can present major therapeutic limitations [20]. Newer, second-generation antipsychotics (SGAs) are accompanied by fewer EPS (~15%) when compared to FGAs (50‒75%) [7,21], but are more frequently associated with adverse metabolic effects [6]. Like FGAs, SGAs block D_2_ receptors; however, they additionally exhibit activity at several serotonin (5-HT) receptors [19,22]. Assorted data suggest an important role of 5-HT neurons and various 5-HT receptors in the modulation of dopaminergic function, and consequently development of EPS following treatment with antipsychotic drugs [23,24,25]. It is possible that molecular determinants of the 5-HT system contribute to the inter-individual differences in development of EPS following treatment with FGAs.

Several studies have reported significant associations between 5-HT receptor gene polymorphisms and the risk of developing EPS [26,27]. Most of these studies have, however, focused on tardive dyskinesia, and only a few investigated the development of acute EPS [28,29]. Therefore, the aim of this study was to investigate the potential relationship of several polymorphisms located in the *HTR1A, HTR1B, HTR2A, HTR2C* and *HTR6* genes, which code for the corresponding 5-HT receptors, with the development of acute EPS, following haloperidol monotherapy. These genetic variants might be clinically useful as pharmacogenetic markers for prediction of the occurrence of acute EPS among patients treated with antipsychotic drugs, as well as for tailoring future genotype-based personalized drug treatments in order to help minimize EPS [30].

## 2. Results

For this study, 299 male patients with schizophrenia were enrolled, with a mean age of 36.49 ± 10.40 years old. Demographic and clinical features of the subjects enrolled are presented in Table 1. Most of the schizophrenia patients had graduated from high school, were unemployed or retired, as well as being either single or divorced and without children. They were also mostly overweight, with a mean BMI of 26.54 ± 9.18 (Table 1). A relatively high proportion of patients were smokers and drank alcohol, while a significant number of patients had previously consumed one or more illegal psychoactive substances. A considerable number of the patients had also previously attempted suicide. As shown in the Table 1, most of the schizophrenia patients previously received antipsychotic therapy (89.52%), usually a combination of typical and atypical antipsychotics (69.00%), whereas a smaller number of the subjects enrolled were drug naïve (10.48%). A majority of the patients previously met the criteria for complete or partial disease remission, whereas 13.10% of them were considered to be treatment-resistant (Table 1). As demonstrated by their high baseline positive, negative, general psychopathology and total Positive and Negative Syndrome Scale (PANSS) scores, all subjects were admitted to the hospital due to acute exacerbation of schizophrenia and subsequently treated with haloperidol. A total of 66.81% of the schizophrenia patients reported some kind of EPS, and in those patients acute EPS usually developed on the 5th day of haloperidol monotherapy.

The EPS were evaluated using the Simpson–Angus Rating Scale for Extrapyramidal Side Effects (SAS), the Barnes Akathisia Rating Scale (BARS) and the Extrapyramidal Symptom Rating Scale (ESRS) (Table 2). According to the SAS scale, some EPS were present in 63.32% of patients, with mean total scores of 4.965 ± 5.643; however, in 111 (48.47%) patients, the acute EPS that appeared following haloperidol treatment were significant (defined as a SAS score > 3), whereas 118 (51.53%) patients were in the group without significant acute EPS (SAS score ≤ 3). As shown in Table 2, according to the SAS ratings, the most frequent and severe EPS were tremors, abnormal gait and excessive salivation. The frequency of akathisia, characterized by a feeling of inner restlessness and an inability to stay still [31], as assessed using the SAS scale, was ~23%. This is consistent with the results (~23%) obtained using the BARS scale to rate akathisia and is also in agreement with the reported rates (5–75%) [32,33] and average prevalence (20–35%) [34] of akathisia. The total mean BARS scores of the schizophrenia patients were 1.489 ± 2.989 (Table 2).

For evaluation using the ESRS scale, some items assessing chronic EPS were excluded from the rating. In the ESRS questionnaire and behavioral scale, 65.50% of patients reported parkinsonism, dystonia or akathisia. The ESRS physician’s examination identified bradykinesia, abnormal gait and posture, as well as rigidity as the most frequent EPS, whereas tremor and rigidity were the most severe EPS based on the highest ESRS scores (Table 2). In concurrence with the SAS and BARS scales, the frequency of akathisia occurrence as assessed by the ESRS scale was ~23%. Although the ESRS clinical global impression detected some symptoms of parkinsonism in ~58% of the subjects, these symptoms were very mild (2.077 ± 1.905 scores), indicating minimal or low-stage parkinsonism (Table 2). The total mean ESRS scores of the schizophrenia patients enrolled was 21.49 ± 21.24. As shown in Table 2, the severity of EPS in schizophrenia patients following haloperidol monotherapy is quite variable, as demonstrated by the high standard deviation (SD) in the BARS, SAS and ESRS scores, and it is probably due to the influence of various environmental as well as genetic factors.

The molecular approach involving gene polymorphisms was studied using real-time PCR, following extraction of DNA from the blood of patients. We focused on the *HTR1A, HTR1B, HTR2A, HTR2C* and *HTR6* gene polymorphisms. The genotype distributions in schizophrenia patients for all of the 5-HT receptor gene polymorphisms tested in the study were in Hardy–Weinberg equilibrium (HWE). As shown in Table 3, no significant differences were observed in the frequency of the genotypes or alleles for any of the 5-HT receptor gene polymorphisms studied between patients with or without significant acute EPS (SAS score >3) following haloperidol monotherapy. However, when we compared the SAS, BARS and ESRS total scores in schizophrenia patients carrying different genotypes or alleles of the 5-HT receptor gene polymorphisms, we found a significant association of the *HTR1B* rs13212041 polymorphism with the total BARS scores (Table 4). Specifically, the total BARS scores were significantly different between patients carrying various *HTR1B* rs13212041 genotypes (*p* = 0.009; Kruskal–Wallis test). Applying of a post-hoc Dunn’s multiple comparisons test demonstrated that haloperidol-treated schizophrenia patients carrying the *HTR1B* rs13212041 TT genotype had a significantly higher total BARS scores (*p* = 0.007) than carriers of the CT genotype.

**Figure 1:**
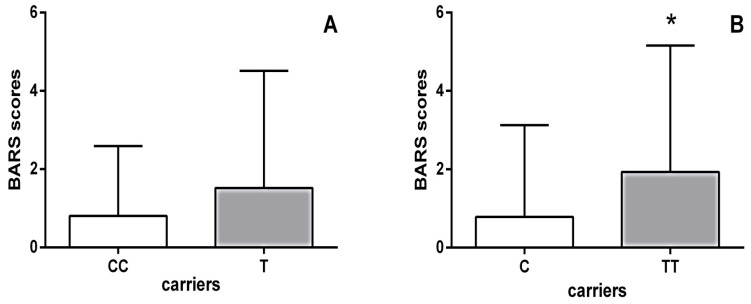
BARS scores of haloperidol-treated schizophrenia patients subdivided according to their *HTR1B* rs13212041 polymorphism status: (**A**) carriers of the homozygous CC genotype (0.800 ± 1.789) vs. carriers of the T allele (1.517 ± 2.996); (**B**) carriers of the homozygous TT genotype (1.931 ± 3.228) vs. carriers of the C allele (0.785 ± 2.341). * *p* = 0.002; Mann–Whitney test, TT vs. C carriers.

Moreover, schizophrenia patients carrying the *HTR1B* homozygous TT genotype had significantly higher BARS scores (*p* = 0.002; Mann–Whitney test) than carriers of the C allele (Figure 1). As shown in Table 5, further analysis using the Kruskal–Wallis test revealed significant differences in the scores of all individual items on the BARS scale between haloperidol-treated schizophrenia patients carrying different genotypes of the *HTR1B* rs13212041 polymorphism. Specifically, Dunn’s multiple comparisons test demonstrated that carriers of the TT genotype had significantly higher scores for each individual item on the BARS score than patients carrying the CT genotype (Table 5).

Since we have found a significant association of the *HTR1B* rs13212041 polymorphism with scores on the BARS scale, which is used for rating akathisia, we evaluated akathisia in haloperidol-treated patients with schizophrenia using the SAS and ESRS scales as well. The results obtained by the Kruskal–Wallis test demonstrated that patients carrying the CC, CT and TT genotypes differed significantly (*p* = 0.008) in severity of akathisia, as assessed using the SAS scale.

Carriers of the *HTR1B* TT genotype (0.519 ± 0.862) also had significantly higher SAS scores for akathisia when compared to carriers of the CT genotype (0.203 ± 0:619) (*p* = 0.006; Dunn’s multiple comparisons test), as well as to carriers of C the allele (0.215 ± 0.634) (*p* = 0.002, Mann–Whitney test). Similarly, when akathisia was evaluated using the ESRS scale, we observed significant differences in the scores between haloperidol-treated schizophrenia patients carrying different *HTR1B* rs13212041 genotypes (*p* = 0.011; Kruskal–Wallis test). Further analysis confirmed that the TT carriers (0.661 ± 1.163) had higher ESRS scores for akathisia when compared to the CT carriers (0.222 ± 0.676; *p* = 0.009; Dunn’s multiple comparisons test), as well as to C-allele carriers (0.221 ± 0.661; *p* = 0.003; Mann–Whitney test). Additionally, TT carriers were significantly more frequent (*p* = 0.006, χ^2^-test) among haloperidol-treated patients with akathisia (81.63%) than in the group without akathisia symptoms (56.52%), as determined by both the SAS and BARS scales. According to the ESRS scale, there were also significantly more schizophrenia patients carrying the TT genotype (*p* = 0.016; χ^2^-test) who developed akathisia (79.59%) than those without akathisia symptoms (57.14%) following haloperidol monotherapy.

## 3. Discussion

To the best of our knowledge, our study is the first to report an association between the *HTR1B* rs13212041 polymorphism and antipsychotic-induced acute EPS, and more specifically akathisia, in schizophrenia patients. Evaluation with the SAS, BARS and ESRS scales revealed a significantly higher frequency of *HTR1B* TT carriers among haloperidol-treated patients who developed akathisia than in those that did not, as well as a higher severity of akathisia in patients carrying the TT genotype in comparison to C-allele carriers. This finding confirms the close link between molecular events affecting the 5-HT system of a patient and their genetic susceptibility to develop antipsychotic-induced EPS [35]. The majority of previous pharmacogenetic studies have focused on chronic EPS, such as tardive dyskinesia [26,27,36,37,38], while only a few reported associations of antipsychotic-induced acute EPS, such as parkinsonism and akathisia, with polymorphisms located in the *HTR2A* and *HTR2C* genes [28,29,39]. In contrast to those findings, our study did not detect any significant molecular associations between the *HTR1A, HTR2A, HTR2C* or *HTR6* gene polymorphisms and acute EPS following haloperidol monotherapy.

**Figure 2:**
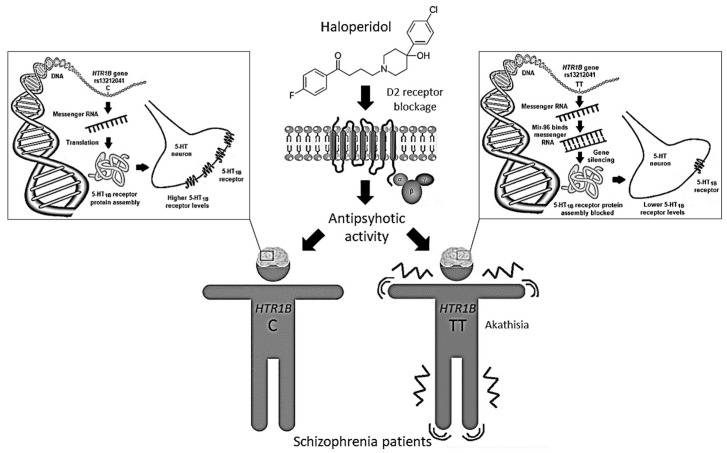
The rs13212041 *HTR1B* gene polymorphism located in the distal 3′-UTR of messenger RNA disrupts the binding site for the microRNA, miR-96, influencing 5-HT_1B_ receptor expression. Haloperidol-treated schizophrenia patients carrying the *HTR1B* TT genotype, who have lower levels of 5-HT_1B_ receptors, develop akathisia more frequently and severely than carriers of the C-allele.

The *HTR1B* rs13212041 (A1997G) polymorphism is located in the distal 3′-untranslational region (UTR) of *HTR1B* messenger RNA, and disrupts the binding site for the microRNA, miR-96, consequently influencing the expression of the 5-HT_1B_ receptor [40]. Expression of miR-96 in the brain [41] may be modulated by various environmental factors [40], including antipsychotic drugs. Carriers of the A-allele show reduced *HTR1B* expression compared to G-allele carriers [40]. Therefore, we can presume that haloperidol-treated schizophrenia patients carrying the TT genotype, who develop akathisia both more frequently and more severely, have lower levels of 5-HT_1B_ receptors than carriers of the C-allele (Figure 2). Such epigenetic mechanisms are supported by the observed associations between DNA methylation patterns in some 5-HT gene promoter regions and response to antipsychotic drugs [42]. Haloperidol was seen to cause an increase in global DNA methylation [43,44], histone 3 phospho-acetylation [45] and expression of various epigenetic modifiers [43]. Moreover, treatment with haloperidol has been associated with altered expression of several miRNAs [43,46,47] and genes [48], some of which may be involved in the development of EPS [49].

The molecular mechanisms by which 5-HT_1B_ receptors might play a role in the development of haloperidol-induced akathisia are elusive. The majority of current antipsychotics act as antagonists at 5-HT_1B_ receptors, and usually demonstrate inverse agonist properties [50,51]. Therefore, the different potencies of individual antipsychotics at 5-HT_1B_ sites, in comparison to D_2_ receptors, could influence their individual propensity to induce EPS. Data regarding the role of 5-HT_1B_ receptors on striatal dopamine release are contradictory [23,52,53]. Instead, 5-HT_1B_ receptors, expressed by striatal cells, would modulate the impact of nigrostriatal dopamine specifically on dopamine-receptive cells of the striatum, independently of the net effect on dopamine efflux [24,54]. This is in line with the findings that 5-HT_1B_ receptor stimulation diminishes the dyskinesia induced by dopamine receptor agonists [55,56]. 5-HT_1B_ receptors act as inhibitory autoreceptors or heteroreceptors on both serotonergic and non-serotonergic neurons, and modulate 5-HT activity [57]. Therefore, the *HTR1B* rs13212041 polymorphism, by influencing 5-HT_1B_ receptor expression, can affect 5-HT neurotransmission, as well as development of antipsychotic-induced EPS.

In our study, all patients were treated with haloperidol as a monotherapy for two weeks, to exclude drug–drug interactions. However, we did not measure haloperidol plasma concentration, nor perform *CYP2D6* genotyping. Haloperidol pharmacokinetics is primarily influenced by the metabolic capacity of the genetically regulated CYP2D6 enzyme [12]. Since antipsychotic dose is a well-known risk factor for EPS, it is possible that patients who carry genotypes associated with poor *CYP2D6* metabolism are at an increased risk of haloperidol-induced EPS [13]. In addition to haloperidol, all patients received diazepam as a concomitant medication for the treatment of agitation, insomnia and anxiety. We cannot, therefore, completely rule out a possible effect of diazepam on haloperidol-induced EPS [58,59]. Although it has been demonstrated that men and women with schizophrenia differ in their treatment response and antipsychotic side effects [60,61], our study enrolled only male schizophrenia patients. As a previous study also found significant gender differences in allele frequencies of the *HRT1B* polymorphism (rs1778258) [62], future studies investigating associations between 5-HT receptor gene variants and EPS should include, as well as compare, male and female patients with schizophrenia. The study of Xia et al. [62] additionally observed different *HRT1B* allelic distributions between schizophrenia patients and healthy control individuals of Han Chinese descent. However, as far as we are aware, this association has not been reported in Caucasian subjects, suggesting ethnicity-related differences. Hence, in addition to an appropriate sample size and statistical power, a significant advantage of the present study is that it involved an ethnically homogenous group of middle-aged male schizophrenia patients in the acute episode of illness. Nevertheless, the lack of healthy control subjects in our study limits its interpretation. Another study limitation is a lack of replication of our findings in an independent sample.

Although akathisia occurs more frequently following the use of high-potency FGAs, such as haloperidol (15–40%), its development has been also observed with certain SGAs [63,64]. Since antipsychotic drug type has been identified as a risk factor for akathisia [31], further studies should test a wider range of antipsychotics for the association observed between the *HRT1B* polymorphism and akathisia. Our results could be of further importance if we consider that akathisia is not limited to antipsychotic medication. Antidepressants, especially selective serotonin reuptake inhibitors (SSRI) [65], monoamine oxidase inhibitors (MAOI) [66] and tricyclic antidepressants (TCA) [67], have also been associated with akathisia. Therefore, if confirmed, the *HTR1B* rs13212041 polymorphism could be a pharmacogenetic predictor of akathisia, to allow better selection of pharmacotherapy and reduction of EPS, resulting in better patient compliance and quality of life. As severe akathisia symptoms can lead to poor adherence to medications, exacerbation of psychiatric symptoms as well as aggression, violence and suicide [68], it is not surprising that interventions aimed at modulating 5-HT transmission have gathered increasing attention for treatment of akathisia [32,69]. The 5-HT_2A/C_ receptor antagonists, mianserin [70], mirtazapine [71], ritanserin [72] and cyproheptadine [73], have all shown some efficacy against acute akathisia. The results of our study suggest that 5-HT_1B_ receptor agonists, such as zolmitriptan, might also be effective as akathisia treatment [74]. Further research is needed, however, in order to verify our finding that the *HTR1B* gene polymorphism is a molecular determinant for developing akathisia, and to further expand our understanding of individual patient susceptibility to EPS induced by various medications.

## 4. Materials and Methods

### 4.1. Subjects and Clinical Evaluation

The study enrolled 229 male patients with schizophrenia recruited from the Psychiatric Hospital Popovaca and the Department of Psychiatry, University Hospital Centre Zagreb, Croatia. The subjects were all admitted to the hospitals due to acute schizophrenia exacerbation. The diagnosis of schizophrenia was made based on the Diagnostic and Statistical Manual of Mental Disorders, Fourth Edition (DSM-IV) criteria [75]. The severity of schizophrenia symptoms was evaluated by experienced psychiatrists using the Positive and Negative Syndrome Scale (PANSS) [76]. The study exclusion criteria were serious somatic illnesses, neurologic disorders and a history of drug use during the previous 6 months. All of the patients enrolled had been without previous antipsychotic medication for at least 48 h. Most of the subjects had not taken antipsychotics for several months and some of them were drug naïve. Patients were treated with haloperidol (15 mg/day, orally or intramuscularly) for two weeks, and adjuvant diazepam therapy (40 mg daily) was introduced in the case of agitation, insomnia and anxiety.

The Simpson–Angus Rating Scale for Extrapyramidal Side Effects (SAS) [77], the Barnes Akathisia Rating Scale (BARS) [78] and the Extrapyramidal Symptom Rating Scale (ESRS) [79] were used to evaluate the severity of EPS during treatment with haloperidol in patients with schizophrenia. As in previous studies [16,29], EPS were defined as significant when SAS scores were > 3, and patients were subsequently subdivided into those with significant acute EPS (SAS score > 3) and those without significant acute EPS (SAS score < 3) following haloperidol monotherapy.

The following items were excluded from the ESRS scale: from part I “Parkinsonism, Dystonia, Dyskinesia and Akathisia—Questionnaire and Behavioral Scale, item 10 (Abnormal involuntary movements (dyskinesia) of extremities or trunk) and item 11 (Abnormal involuntary movements (dyskinesia) of tongue, jaw, lips or face); from part III “Dystonia: Physician´s examination”: item 2 (Non-acute or chronic or tardive dystonia); and all of part IV “Dyskinetic movements: Physician´s examination” and of part V “Clinical global impression of severity of Dyskinesia”.

This study was approved by the ethics committees of the Psychiatric Hospital Popovaca and of the University Hospital Centre Zagreb, and was carried out in accordance with the Declaration of Helsinki, 1996 (and its amendments). All participants were Caucasians living in Croatia. Only patients who provided signed informed consent were included in the study.

### 4.2. Blood Collection and Genotyping

Samples of blood (4 mL) from patients with schizophrenia were collected using a plastic syringe containing 1 mL anticoagulant (acid citrate dextrose). Genomic DNA was isolated from peripheral blood leukocytes by a standard salting-out method [80]. Genotyping was performed according to the manufacturer’s protocol (Applied Biosystems), using TaqMan SNP Genotyping Assays and TaqMan Genotyping Master Mix. TaqMan allele-specific polymerase chain reaction (PCR) was conducted on ABI Prism 7000 Sequencing Detection System apparatus. Briefly, 20 ng of genomic DNA was amplified in a 10 µL reaction volume, using these PCR reaction conditions: 40 cycles at 92 °C for 15 s and 60 °C for 60 s. The *HTR1A* rs6295, *HTR1B* rs13212041*, HTR2A* rs6313, *HTR2C* rs3813929 and *HTR6* rs1805054 polymorphisms were analyzed (Table 6).

### 4.3. Data Analyses

Statistical analyses were performed with GraphPad Prism version 4.00 for Windows (GraphPad Software, Inc., San Diego, CA, USA). The data are expressed as number (*n*) and percentage (%) or as mean ± SD. Normality of distribution was assessed with the D’Agostino–Pearson omnibus normality test. Since the data was found not to be normally distributed, the Mann–Whitney U-test was used for comparison of two groups, while the Kruskal–Wallis test and post-hoc Dunn’s multiple comparison test were used for analysis of three groups. Possible deviations from HWE were tested using the goodness of fit χ^2^-test. Genotype and allele frequencies were evaluated by a χ^2^-test of independence or Fisher exact test, respectively. The results were corrected for multiple testing (5 polymorphisms) using Bonferroni correction, and the *p*-value for significance was set to 0.01. G*Power 3 Software was used for conducting power analyses, i.e., to determine a priori sample size and to post hoc compute the power achieved. For analyses with a χ^2-^test (with α = 0.01; power (1−β) = 0.80 and a small effect size (ω = 0.25)), for df = 2, the total desired sample size was 223, and for df = 1 (Fisher exact test), the total desired sample size was 187. For the F test (Kruskal–Wallis test) involving three groups (with α = 0.01; power = 0.80; a small effect size = 0.25), the total desired sample size was 227. For the t-test (Mann–Whitney test) (with α = 0.01; power = 0.80; median effect size = 0.50), total desired sample size was 228. As the actual total sample size was 229, the power analysis confirmed the appropriate sample size and thus statistical power of the study.

## 5. Conclusions

To the best of our knowledge, this is the first study to report an association of the *HTR1B* rs13212041 polymorphism with antipsychotic-induced akathisia. Our results demonstrate that homozygous patients with schizophrenia who carry the TT genotype are more prone to develop akathisia and experience higher akathisia severity following haloperidol therapy than carriers of the C-allele. These molecular findings indicate the potential involvement of 5-HT_1B_ receptors in the development of akathisia in haloperidol-treated patients. As the rs13212041 polymorphism affects microRNA regulation of *HTR1B* gene expression, these data might suggest a role for epigenetic mechanisms in 5-HT modulation associated with antipsychotic-induced EPS. Further studies, including a larger number of subjects, should test a wider range of antipsychotics for association between the *HRT1B* polymorphism and akathisia, and should also include and compare male and female patients with schizophrenia. If confirmed, such pharmacogenetic predictors of EPS could be helpful toward a better selection of medication in order to reduce EPS, resulting in better patient compliance and quality of life. For schizophrenia patients for whom haloperidol remains an important treatment option, 5-HT_1B_ receptor agonists might represent a useful therapeutic approach for management of akathisia.

## Figures and Tables

**Table 1:** Socio-demographic and clinical characteristics of haloperidol-treated schizophrenia patients.

Male Schizophrenia Patients	*n* = 229
Age (years, mean ± SD)	36.49 ± 10.40
BMI (mean ± SD)	26.54 ± 9.18
Secondary education (*n*, %)	163 (71.18%)
Not working (unemployed or retired) (*n*, %)	181 (79.04%)
Not married (*n*, %)	206 (89.96%)
Without children (*n*, %)	192 (83.84%)
Alcohol consumption (*n*, %)	111 (48.47%)
Smoking (*n*, %)	154 (67.25%)
Illegal drug consumption (*n*, %) *	49 (21.40%)
PANSS positive scores (mean ± SD)	35.76 ± 4.74
PANSS negative scores (mean ± SD)	34.41 ± 4.97
PANSS general psychopathology scores (mean ± SD)	61.57 ± 7.40
PANSS total scores (mean ± SD)	131.70 ± 13.12
Suicide attempt (*n*, %)	57 (24.89%)
Previous antipsychotic medication (*n*, %) **	205 (89.52%)
Previous complete or partial disease remission (*n*, %) ***	199 (86.90%)
Time of acute EPS onset after haloperidol therapy (days, mean ± SD)	5.04 ± 2.76
Acute EPS occurrence after haloperidol therapy (*n*, %)	153 (66.81%)

* No drugs during the previous 6 months. ** No previous antipsychotic medication for at least 48 h. *** Remission criteria: Positive and Negative Syndrome Scale (PANSS) items P1, P2, P3, N1, N4, N6, G5, G9 ≤ 3 for at least 6 months.

**Table 2:** Number and percentage (%) of schizophrenia patients with particular acute extrapyramidal side effects (EPS) and its severity (scores), as assessed with the Simpson–Angus Rating Scale for Extrapyramidal Side Effects (SAS), the Barnes Akathisia Rating Scale (BARS) and the Extrapyramidal Symptom Rating Scale (ESRS) scales following haloperidol treatment.

EPS	Number of Patients (%)	Scores (Mean ± SD)
**SAS scale**		
Gait	82 (35.81 %)	0.635 ± 0.936
Arm dropping	68 (29.69%)	0.446 ± 0.758
Shoulder shaking	47 (20.52%)	0.338 ± 0.730
Elbow rigidity	67 (29.26%)	0.459 ± 0.788
Wrist rigidity	66 (28.82%)	0.441 ± 0.763
Head rotation	64 (27.95%)	0.464 ± 0.838
Glabella tap	44 (19.21%)	0.231 ± 0.524
Tremor	87 (37.99%)	0.878 ± 1.193
Salivation	81 (35.37%)	0.790 ± 1.151
Akathisia	53 (23.14%)	0.402 ± 0.798
Total SAS scores	145 (63.32%)	4.965 ± 5.643
Significant acute EPS after haloperidol therapy *	111 (48.47%)	SAS score >3
**BARS scale**		
Objective	52 (22.71%)	0.367 ± 0.735
Subjective: Awareness of restlessness	52 (22.71%)	0.375 ± 0.777
Subjective: Distress related to restlessness	51 (23.14%)	0.367 ± 0.770
Global clinical assessment of akathisia	53 (23.14%)	0.377 ± 0.755
Total BARS scores	53 (23.14%)	1.489 ± 2.989
ESRS scale **		
I. Parkinsonism, dystonia, dyskinesia and akathisia —questionnaire and behavioral scale	150 (65.50%)	5.018 ± 4.774
II. Parkinsonism and akathisia exam—physician’s examination		
Expressive automatic movements (facial mask/speech)	90 (39.30%)	0.797 ± 1.097
Bradykinesia	99 (43.23%)	0.860 ± 1.143
Rigidity	91 (39.74%)	3.081 ± 4.333
Gait and posture	95 (41.48%)	0.851 ± 1.185
Tremor	79 (34.50%)	3.383 ± 5.198
Akathisia	54 (23.58%)	0.495 ± 1.015
Sialorrhea	76 (33.19%)	1.054 ± 1.521
Postural stability	58 (25.33%)	0.423 ± 0.824
III. Dystonia—physician´s examination—1. Acute torsion dystonia	40 (17.47%)	1.680 ± 4.745
VI. Clinical global impression of severity of parkinsonism	135 (58.95%)	2.077 ± 1.905
VII. Clinical global impression of severity of dystonia	44 (19.21%)	0.874 ± 1.889
VIII. Stage of parkinsonism	132 (57.64%)	1.473 ± 1.334
Total ESRS scores	150 (65.50%)	21.49 ± 21.24

* Patients with SAS score > 3 are considered to have significant acute EPS. ** In the ESRS scale the following were excluded: items 10 and 11 from part I, item 2 from part III and parts IV and V.

**Table 3:** Genotype and allele frequencies of 5-HT receptor gene polymorphisms in schizophrenia patients, subdivided according to the development of significant acute EPS following haloperidol therapy.

SNP	Genotype (*n*, %)			χ^2^-Test	Allele (*n*, %)		Fisher’s Test
***HTR2A*** **rs6313**	**CC**	**CT**	**TT**	*p* = 0.183	**C**	**T**	*p* = 0.183
Significant acute EPS *	32 (28.83%)	60 (54.05%)	19 (17.12%)		120 (55.05%)	98 (44.95%)	
No significant acute EPS	47 (39.83%)	51 (43.22%)	20 (16.95%)		145 (61.44%)	91 (38.56%)	
***HTR2C*** rs3813929**					**C**	**T**	*p* = 0.510
Significant acute EPS *					91 (81.98%)	20 (18.02%)	
No significant acute EPS					92 (77.97%)	26 (22:03%)	
***HTR1A*** **rs6295**	**CC**	**CG**	**GG**	*p* = 0.585	**C**	**G**	*p* = 0.433
Significant acute EPS *	30 (29.70%)	51 (50.50%)	20 (19.80%)		111 (54.95%)	91 (45.05%)	
No significant acute EPS	25 (23.36%)	59 (55.14%)	23 (21.50%)		109 (50.93%)	105 (49.07%)	
***HTR1B*** **rs13212041**	**CC**	**CT**	**TT**	*p* = 0.055	**C**	**T**	*p* = 0.330
Significant acute EPS *	4 (3.88%)	29 (28.15%)	70 (67.96%)		37 (17.96%)	169 (82.04%)	
No significant acute EPS	1 (0.93%)	45 (42.06%)	61 (57.01%)		47 (21.96%)	167 (78.04%)	
***HTR6*** **rs1805054**	**CC**	**CT**	**TT**	*p* = 0.246	**C**	**T**	*p* = 0.120
Significant acute EPS *	55 (55.56%)	39 (39.39%)	5 (5.05%)		149 (75.25%)	49 (24.75%)	
No significant acute EPS	73 (66.36%)	34 (30.91%)	3 (2.73%)		180 (81.82%)	40 (18.18%)	

* Patients with SAS score >3 are considered to have significant acute EPS. ** Since the *HTR2C* gene is located on the X chromosome, for the rs3813929 polymorphism only allele frequencies are available.

**Table 4:** SAS, BARS and ESRS total scores in haloperidol-treated schizophrenia patients carrying different genotypes or alleles of 5-HT receptor gene polymorphisms.

SNP	Genotype/Allele			Statistics
***HTR2A*** **rs6313**	**CC**	**CT**	**TT**	
SAS score (mean ± SD)	4.177 ± 5.257	5.802 ± 6.145	4.179 ± 4.588	*p* = 0.111; Kruskal–Wallis test
BARS score (mean ± SD)	1.304 ± 2.695	1.595 ± 3:203	1.564 ± 2.981	*p* = 0.857; Kruskal–Wallis test
ESRS score (mean ± SD)	19.22 ± 21.13	24.62 ± 22.16	17.10 ± 17.62	*p* = 0.083; Kruskal–Wallis test
***HTR2C*** **rs3813929**	**/**	**C**	**T**	
SAS score (mean ± SD)		4.951 ± 5.502	5.022 ± 6.238	*p* = 0.702; Mann–Whitney test
BARS score (mean ± SD)		1.596 ± 3:094	1.065 ± 2.516	*p* = 0.294; Mann–Whitney test
ESRS score (mean ± SD)		21.96 ± 21.26	19.63 ± 21.32	*p* = 0.364; Mann–Whitney test
***HTR1A*** **rs6295**	**CC**	**CG**	**GG**	
SAS score (mean ± SD)	5.491 ± 5.316	4.682 ± 5.525	4.814 ± 6.013	*p* = 0.451; Kruskal–Wallis test
BARS score (mean ± SD)	1.927 ± 3.271	1.655 ± 3.303	0.953 ± 2.104	*p* = 0.393; Kruskal–Wallis test
ESRS score (mean ± SD)	21.26 ± 19.37	22.03 ± 21.37	21.29 ± 23.00	*p* = 0.962; Kruskal–Wallis test
***HTR1B*** **rs13212041**	**CC**	**CT**	**TT**	
SAS score (mean ± SD)	6.200 ± 4.764	3.797 ± 4.728	5.580 ± 6.027	*p* = 0.077; Kruskal–Wallis test
BARS score (mean ± SD)	0.800 ± 1.789	0.7838 ± 2.383	1.931 ± 3.228	*p* = 0.009; Kruskal–Wallis test *
ESRS score (mean ± SD)	22.40 ± 17.40	16.92 ± 17.51	24.78 ± 23.22	*p* = 0.089; Kruskal–Wallis test
***HTR6*** **rs1805054**	**CC**	**CT**	**TT**	
SAS score (mean ± SD)	4.359 ± 5.246	5.178 ± 5.414	9.143 ± 8.275	*p* = 0.094; Kruskal–Wallis test
BARS score (mean ± SD)	1.617 ± 2.986	1.315 ± 2.990	2.000 ± 4.276	*p* = 0.513; Kruskal–Wallis test
ESRS score (mean ± SD)	19.68 ± 19.79	24.04 ± 23.13	26.13 ± 20.93	*p* = 0.340; Kruskal–Wallis test

* *p* = 0.007 using Dunn’s multiple comparisons test, CT vs. TT carriers.

**Table 5:** Scores of individual BARS items in haloperidol-treated schizophrenia patients carrying different genotypes of the *HTR1B* rs13212041 polymorphism.

*HTR1B* rs13212041	Genotypes			Statistics
BARS Scale Scores	CC	CT	TT	Kruskal–Wallis Test
Objective (mean ± SD)	0.200 ± 0.447	0.189 ± 0.589	0.481 ± 0.798	*p* = 0.010 ^a^
Subjective: Awareness of restlessness (mean ± SD)	0.200 ± 0.447	0.203 ± 0.619	0.481 ± 0.844	*p* = 0.012 ^b^
Subjective: Distress related to restlessness (mean ± SD)	0.200 ± 0.447	0.203 ± 0.619	0.473 ± 0.844	*p* = 0.016 ^c^
Global clinical assessment of akathisia (mean ± SD)	0.200 ± 0.447	0.189 ± 0.589	0.500 ± 0.828	*p* = 0.007^d^

^a^*p* = 0.007, ^b^ *p* = 0.009, ^c^ *p* = 0.013 and ^d^ *p* = 0.005 using Dunn’s multiple comparisons test, TT vs. CT carriers.

**Table 6:** Details of the 5-HT receptor gene polymorphisms analyzed in the study.

SNP ID	Assay ID	Location	SNP Type	Context Sequence [VIC/FAM]
***HTR2A*** **rs6313**	C___3042197_1_	Chr. 13: 46895805 on GRCh38	Intron, Transition Substitution, Silent Mutation, Intragenic	ATGCATCAGAAGTGTTAGCTTCTCC[A/G]GAGTTAAAGTCATTACTGTAGAGCC
***HTR2C*** **rs3813929**	C__27488117_10	Chr. X: 114584047 on GRCh38	Transition Substitution, Intron, Intragenic	CTGCTCTTGGCTCCTCCCCTCATCC[C/T]GCTTTTGGCCCAAGAGCGTGGTGCA
***HTR1A*** **rs6295**	C__11904666_10	Chr. 5: 63962738 on GRCh38	Intron, Transversion Substitution, Intragenic	ATGGAAGAAGACCGAGTGTGTCTTC[C/G]TTTTTAAAAAGCTACCTCCGTTCTC
***HTR1B*** **rs13212041**	C__32252506_10	Chr. 6: 77461407 on GRCh38	Transition Substitution, UTR 3, Intragenic	AAAAAATAAAGCAGTCTGCAGACTT[C/T]GGCACTAGCACACATAATGGTTTGT
***HTR6*** **rs1805054**	C___1264819_10	Chr. 1: 19666020 on GRCh38	Transition Substitution, Silent Mutation, Intragenic	CGCCGGCCATGCTGAACGCGCTGTA[C/T]GGGCGCTGGGTGCTGGCGCGCGGCC

## Abbreviations

BARS	Barnes Akathisia Rating Scale
BMI	Body mass index
DSM-IV	Diagnostic and Statistical Manual of Mental Disorders, Fourth Edition
EPS	Extrapyramidal side effects
ESRS	Extrapyramidal Symptom Rating Scale
FGAs	Typical or first-generation antipsychotics
5-HT	5-hydroxytryptamine; Serotonin
HWE	Hardy–Weinberg equilibrium
PANSS	Positive and Negative Syndrome Scale
PCR	Polymerase chain reaction
SAS	Simpson–Angus Rating Scale for Extrapyramidal Side Effects
SGAs	Atypical or second-generation antipsychotics
WHO	World Health Organization
